# A Novel Lysophosphatidic Acid Acyltransferase of *Escherichia coli* Produces Membrane Phospholipids with a *cis*-vaccenoyl Group and Is Related to Flagellar Formation

**DOI:** 10.3390/biom10050745

**Published:** 2020-05-11

**Authors:** Yosuke Toyotake, Masayoshi Nishiyama, Fumiaki Yokoyama, Takuya Ogawa, Jun Kawamoto, Tatsuo Kurihara

**Affiliations:** 1 Institute for Chemical Research, Kyoto University, Uji, Kyoto 611-0011, Japan; toyotake@fc.ritsumei.ac.jp (Y.T.); mnishiyama@phys.kindai.ac.jp (M.N.); yokoyama@mbc.kuicr.kyoto-u.ac.jp (F.Y.); ogawa.tky@mbc.kuicr.kyoto-u.ac.jp (T.O.); jun_k@mbc.kuicr.kyoto-u.ac.jp (J.K.); 2 Department of Biotechnology, College of Life Sciences, Ritsumeikan University, 1-1-1 Noji-higashi, Kusatsu, Shiga 525-8577, Japan

**Keywords:** lysophosphatidic acid acyltransferase, membrane phospholipid diversity, swimming motility, flagellar formation

## Abstract

Lysophosphatidic acid acyltransferase (LPAAT) introduces fatty acyl groups into the *sn*-2 position of membrane phospholipids (PLs). Various bacteria produce multiple LPAATs, whereas it is believed that *Escherichia coli* produces only one essential LPAAT homolog, PlsC—the deletion of which is lethal. However, we found that *E. coli* possesses another LPAAT homolog named YihG. Here, we show that overexpression of YihG in *E. coli* carrying a temperature-sensitive mutation in *plsC* allowed its growth at non-permissive temperatures. Analysis of the fatty acyl composition of PLs from the *yihG*-deletion mutant (∆*yihG*) revealed that endogenous YihG introduces the *cis*-vaccenoyl group into the *sn*-2 position of PLs. Loss of YihG did not affect cell growth or morphology, but ∆*yihG* cells swam well in liquid medium in contrast to wild-type cells. Immunoblot analysis showed that FliC was highly expressed in ∆*yihG* cells, and this phenotype was suppressed by expression of recombinant YihG in ∆*yihG* cells. Transmission electron microscopy confirmed that the flagellar structure was observed only in ∆*yihG* cells. These results suggest that YihG has specific functions related to flagellar formation through modulation of the fatty acyl composition of membrane PLs.

## 1. Introduction

Phospholipids (PLs) are the primary component of biological membranes. They consist of a phosphate-containing head group and two fatty acyl groups. The structural diversity of these fatty acyl groups affects the physical state of biological membranes such as fluidity, permeability, rigidity, and thickness [1,2,3,4]. Bacteria maintain the ideal physical state of their membrane in response to environmental changes by modulating the fatty acyl groups of membrane PLs. For example, as environmental temperature decreases, bacteria generally introduce lower-melting-point fatty acyl groups such as unsaturated fatty acyl groups and branched-chain fatty acyl groups into membrane PLs [5,6,7,8,9,10,11,12,13]. However, it is still not fully understood how bacteria regulate the fatty acyl composition of membrane PLs in response to environmental changes.

During the de novo synthesis of PLs [14,15,16,17], fatty acyl groups are incorporated into the *sn*-1 and *sn*-2 position by glycerol-3-phosphate acyltransferases and lysophosphatidic acid acyltransferases (LPAATs), respectively [18,19,20,21,22,23]. In some bacteria, such as *Neisseria meningitidis*, *Pseudomonas fluorescens*, *Pseudomonas aeruginosa*, and *Rhodobacter capsulatus*, multiple LPAAT homologs have been identified and characterized [24,25,26,27]. These LPAATs potentially play different roles in vivo to contribute to the generation of diversity in membrane PLs.

*Shewanella livingstonensis* Ac10 is a psychrotrophic bacterium used as a model of cold-adapted organisms [9,28,29,30,31]. This bacterium has five LPAAT homologs (SlPlsC1 to SlPlsC5) [32]. We previously reported that SlPlsC1 plays a major role in the synthesis of PLs containing polyunsaturated fatty acyl groups [32,33], while SlPlsC4 is mainly responsible for the synthesis of PLs containing branched-chain fatty acyl groups (i13:0 and i15:0) [34]. Some marine bacteria such as *Alteromonas mediterranea* and *Colwellia psychrerythraea* have a putative SlPlsC4 ortholog. These bacteria also have an SlPlsC1 ortholog, suggesting that the multiple LPAAT homologs introduce specific fatty acyl groups into membrane PLs for their adaptation to the marine environment, as has been shown in *S. livingstonensis* Ac10. Likewise, it is conceivable that uncharacterized LPAAT homologs also exist in other bacterial species to generate membrane lipid diversity to allow environmental adaptation.

It has long been believed that *Escherichia coli* has only one essential LPAAT homolog, named PlsC—the deletion of which is lethal [35]. However, we found that *E. coli* possesses an SlPlsC4 ortholog named YihG. YihG was originally thought to be a second poly(A) polymerase [36], but this claim has subsequently been denied [37]. Even though YihG can be considered as an inner membrane protein belonging to a 1-acyl-*sn*-glycerol-3-phosphate *O*-acyltransferase family based on its conserved catalytic motif [38,39], the sequence identity between YihG and *E. coli* PlsC is 17.9%, and thus YihG has not been recognized as a functional LPAAT homolog. Sutton and co-workers previously reported that overproduction of YihG suppresses the hyperinitiation of DNA replication and resulting growth defect in *E. coli*, presumably by regulating the level of ATP-binding DnaA (DnaA-ATP), an activator for the initiation of DNA replication [40]. However, no one has yet clarified whether YihG has LPAAT activity or investigated how YihG affects the membrane lipid composition. YihG is conserved in some γ-proteobacteria such as *P. aeruginosa*, *Salmonella typhimurium*, and *Vibrio cholerae*. Thus, we hypothesized that an SlPlsC4 ortholog has a physiological function in various γ-proteobacteria including some enteric and pathogenic bacteria.

In this study, we demonstrated that YihG is a functional LPAAT homolog by complementation assay using the *E. coli* strain JC201, a temperature-sensitive *plsC* mutant. We found that YihG has a different substrate specificity from PlsC, and that endogenous YihG contributes to the synthesis of PLs containing a *cis*-vaccenoyl group at the *sn*-2 position. The lack of YihG caused enhanced flagellar formation and swimming motility in the liquid medium. Thus, *E. coli* YihG appears to regulate bacterial swimming motility through modulation of the composition of fatty acyl groups in membrane PLs.

## 2. Materials and Methods

### 2.1. Bacterial Strains, Plasmids, and Growth Conditions

The bacterial strains and plasmids used in this study are listed in Table S1. *E. coli* K-12 strain BW25113 and its *yihG*-knockout mutant (∆*yihG*) were obtained from the National BioResource Project (NIG, Mishima, Japan). *E. coli* JC201 strain, which carries a temperature-sensitive mutation in *plsC*, was used in the complementation assay to evaluate LPAAT activity [35]. The *E. coli* cells were cultivated in lysogeny broth [LB; 1% (*w*/*v*) Bacto Tryptone, 0.5% (*w*/*v*) Bacto Yeast extract, and 1% (*w*/*v*) NaCl], tryptone broth [TB; 1% (*w*/*v*) Bacto Tryptone and 1% (*w*/*v*) NaCl], and M9-based minimal medium [48 mM Na_2_HPO_4_, 22 mM KH_2_PO_4_, 19 mM NH_4_Cl, 8.6 mM NaCl, 0.1 mM CaCl_2_, 1 mM MgSO_4_, and 0.4% (*w*/*v*) glucose]. Growth was monitored by measuring OD_600_ with a UV–visible spectrophotometer (UV-2450, Shimadzu, Kyoto, Japan). Antibiotics were used, when required, at the following concentrations: kanamycin (30 µg/mL) and chloramphenicol (34 µg/mL).

### 2.2. Construction of YihG- and PlsC-Expression Plasmids

PCR primers used in this study are listed in Table S2. The plasmid named pBAD-Cm^R^ was created from pBAD28 [41] by digestion with ScaI and self-ligation to remove the ampicillin resistance gene. DNA fragments coding for the C-terminal hexa-histidine-tagged YihG (YihG-His_6_) and PlsC (PlsC-His_6_) were obtained by PCR using the BW25113 genome as a template. The resulting DNA fragments were individually introduced into the SalI-HindIII site in pBAD-Cm^R^ using an In-Fusion Advantage PCR cloning kit (TaKaRa Bio, Otsu, Japan) following the manufacturer’s instructions. The resulting plasmids were designated pBAD/*yihG-his_6_* and pBAD/*plsC-his_6_*, respectively.

### 2.3. JC201 Complementation Assay

JC201 cells harboring pBAD-Cm^R^ or pBAD/*yihG-his_6_* were cultured at 30 °C in LB until the OD_600_ reached 1.2 to 1.4. The cell cultures were normalized to an OD_600_ of 1.0 and diluted to 10^−2^, 10^−3^, 10^−4^, 10^−5^, and 10^−6^ in fresh LB. Three microliters of the serial dilutions was spotted onto 1.5% (*w*/*v*) agar LB plates containing 0 to 2% (*w*/*v*) l-arabinose. The plates were incubated at 30 and 42 °C until colonies were formed.

JC201 cells harboring pBAD-Cm^R^, pBAD/*yihG-his_6_*, or pBAD/*plsC-his_6_* were cultured at 30 °C in LB until an OD_600_ reached 1.0 to 1.8. They were diluted to an OD_600_ of 0.01 in LB containing 0.5% (*w*/*v*) and 1% (*w*/*v*) l-arabinose and grown at 37 °C for 9 h to monitor their growth rates. To analyze membrane lipids as described below, cells expressing YihG and PlsC with 1% (*w*/*v*) l-arabinose were grown and harvested by centrifugation at room temperature when their OD_600_ reached 0.6 to 0.8.

### 2.4. Total PL Extraction and Analysis by Electrospray Ionization Tandem Mass Spectrometry (ESI–MS/MS)

BW25113 cells harboring pBAD-Cm^R^ and ∆*yihG* cells harboring pBAD-Cm^R^ or pBAD/*yihG-his_6_* were grown in TB containing 0.2% (*w*/*v*) l-arabinose at 37 °C to an OD_600_ of 0.4 to 0.6 and harvested by centrifugation at room temperature. The cells were flash frozen in liquid nitrogen and stored at −80 °C until use. The frozen cells were lyophilized, and total PLs were extracted using the Bligh and Dyer method [42]. JC201 cells harvested as described above were not lyophilized and were instead directly used for PL extraction. The extracted PLs were analyzed by ESI–MS/MS [a triple-quadrupole Sciex API 3000™ LC/MS/MS System (Applied Biosystems, Foster City, CA, USA)], as described previously [34].

### 2.5. Analysis of the sn-1 and sn-2 Fatty Acyl Groups of PLs

The PLs prepared as described above were hydrolyzed with Phospholipase A2 (PLA2, P6534, Sigma, St. Louis, MO). The resulting *sn*-2 fatty acyl groups were extracted by the Dole’s method [34,43,44] and analyzed by gas chromatography–mass spectrometry [GC–MS, Clarus 680 gas chromatograph interfaced with Clarus SQ 8C mass spectrometer (Perkin Elmer, Wellesley, MA) equipped with an Agilent J&W GC column DB-1 (Agilent Technologies Inc., Santa Clara, CA, USA)], as described previously [45]. Lysophospholipids (LPLs) were analyzed using the total lipid extracts from an aliquot of the PLA2 reaction product by ESI–MS as described above.

### 2.6. Motility Assay in Soft Agar Plates

The BW25113 and ∆*yihG* cells were grown in LB at 37 °C to an OD_600_ of 0.8 to 1.2. Two microliters of each cell culture was spotted on TB 0.2% (*w*/*v*) agar plates. For ∆*yihG* cells harboring the plasmids, TB 0.2% (*w*/*v*) agar plates containing 0.02% (*w*/*v*) or 0.2% (*w*/*v*) l-arabinose were used. The plates were incubated at 37 °C for an appropriate time, as noted in the Results section.

### 2.7. Microscopic Observation of Swimming Cells

The BW25113 and ∆*yihG* cells were grown at 37 °C in TB to an OD_600_ of 0.6 to 1.3. The cultivated cells were diluted with fresh TB medium. Motility of the cells was observed at room temperature under a microscope (Ti–E, Nikon, Tokyo, Japan) [46]. The phase-contrast images of cells near the cover slip were recorded at video rate. All assays were repeated with four different cultures. The fraction of the swimming cells was obtained by dividing the number of cells that swam with a speed of >2 µm/s by that of all cells in the focal plane. The speed of each of the swimming cells was analyzed using a custom-made plugin (Version 0.7.1) of Image J [47].

### 2.8. Flagellin Preparation and Analysis by Western Blot Analysis

The BW25113 and ∆*yihG* cells were grown at 37 °C in TB to an OD_600_ of 0.5 to 0.7. ∆*yihG* cells harboring the plasmids were grown in TB containing 0.2% (*w*/*v*) l-arabinose. The culture was transferred into a tube and vigorously shaken to shear off cell-associated flagella. The sample was centrifuged to spin down the cells, and the supernatant was passed through a filter with a pore size of 0.45 µm. The filtrated supernatant was concentrated with an Amicon Ultra device (30,000 MWCO), and the flagella in the supernatant were precipitated with trichloroacetic acid. The precipitates were subjected to SDS-PAGE and Western blot analysis using the rabbit anti-flagellin antibody [48]. The signal was detected using a peroxidase-conjugated anti-rabbit IgG antibody (Sigma, St. Louis, MO, USA) and Chemi-Lumi One Ultra (Nacalai Tesque, Kyoto, Japan).

### 2.9. Transmission Electron Microscope (TEM) Observation of the Flagellar Structures

TEM observation of the flagellar structures was performed according to the method described by Furuno et al. [49]. The BW25113 and ∆*yihG* cells were grown at 37 °C on TB 1.5% (*w*/*v*) agar plates until colonies were formed. Some colonies were scraped with a toothpick and gently suspended in 2.5 µL of water to prevent detachment of the flagellar from the cells. Two microliters of the suspension was adsorbed onto a hydrophilized carbon-coated copper grid and then treated twice with 2% (*w*/*v*) phosphotungstic acid solution. The TEM images were obtained with a JEM-1400 transmission electron microscope (JEOL, Ltd., Tokyo, Japan) at an accelerating voltage of 120 kV. Images were acquired using a charge-coupled device camera (a built-in camera in the JEM-1400).

## 3. Results

### 3.1. Identification of YihG in E. coli as an SlPlsC4 Ortholog

Analysis of the *E. coli* genome using the BLAST program with the SlPlsC4 amino acid sequence (accession number, BBD74888) as a query revealed that *E. coli* YihG (accession number, AIN34165), a putative membrane acyltransferase, is an SlPlsC4 ortholog. The pairwise sequence alignment using the EMBOSS Needle global alignment tool (https://www.ebi.ac.uk/Tools/psa/emboss_needle/) showed that the amino acid sequence of YihG shares 39.1% identity with that of SlPlsC4. YihG contains highly conserved acyltransferase motifs I–III but does not contain a motif IV like SlPlsC4 (Figure S1) [32,39]. These results suggested that YihG has a similar enzymatic activity to SlPlsC4.

### 3.2. Overexpression of YihG in an E. coli plsC Mutant Allows its Growth at Non-Permissive Temperatures

**Figure 1:**
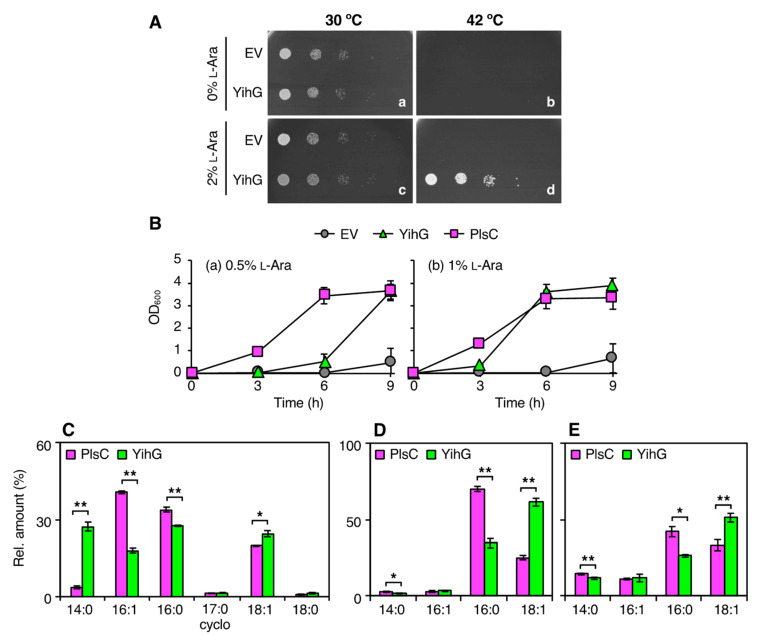
Overexpression of YihG and PlsC in JC201 cells. (**A**) Serially diluted JC201 cells harboring the pBAD-Cm^R^ empty vector (EV) or pBAD/*yihG-his_6_* (YihG) were grown on LB plates containing 0% or 2% l-arabinose at 30 °C (a, c) and 42 °C (b, d) for 12–14 h. (**B**) JC201 cells harboring the pBAD-Cm^R^ empty vector (EV, gray), pBAD/*yihG-his_6_* (YihG, light green), or pBAD/*plsC-his_6_* (PlsC, magenta) were grown in LB containing 0.5% (a) or 1% (b) l-arabinose at 37 °C. Each data point is the average of three biological replicates ± SD. (**C**–**E**) The fatty acyl composition of phospholipids (PLs) from JC201 cells overexpressing YihG and PlsC. Total PL extracts were hydrolyzed by PLA2, and the resulting fatty acids were analyzed by GC–MS (**C**), whereas the resulting lysophosphatidylethanolamines (LPEs) and lysophosphatidylglycerols (LPGs) were analyzed by ESI–MS (D and E, respectively). The graphs show the relative amounts of the fatty acids and lysophospholipids (LPLs) from JC201 cells harboring pBAD/*plsC-his_6_* (PlsC, magenta) or pBAD/*yihG-his_6_* (YihG, light green). The cyclopropane derivative of 16:1 is indicated as 17:0cyclo (**C**). Data are shown as the mean ± SD (*n* = 3). Statistical analysis was performed using Welch’s *t*-test: *, *p* < 0.05; **, *p* < 0.01.

To examine whether YihG is a functional LPAAT homolog, we performed in vivo complementation assays using *E. coli* strain JC201, carrying a temperature-sensitive mutation in *plsC*. This strain grows normally at 30 °C, but not at higher temperatures [35]. The pBAD derivatives, expressing YihG or PlsC upon induction by l-arabinose, were introduced into JC201 cells, and the transformants were tested for their ability to grow at non-permissive temperatures. In plate assays, cells expressing the recombinant YihG grew well at 42 °C in the presence of 2% l-arabinose but showed no or marginal growth in the presence of 0 to 1% l-arabinose (Figure 1A and Figure S2). No complementation was observed in cells harboring the empty vector under the same conditions (Figure 1A and Figure S2). Thus, it was demonstrated that YihG, like PlsC, can act as an LPAAT in vivo.

To investigate the physiological contribution of YihG to cell growth, we compared the growth rates of JC201 cells overexpressing YihG with JC201 cells overexpressing PlsC (Figure 1B). JC201 cells harboring the empty vector hardly grew at 37 °C. However, cells overexpressing PlsC showed vigorous growth at 37 °C in the presence of both 0.5% and 1% l-arabinose. The growth rate of cells overexpressing YihG at 37 °C was much slower than that of cells overexpressing PlsC in the presence of 0.5% l-arabinose, but these strains grew similarly in the presence of 1% l-arabinose. These results indicated that YihG, expressed following l-arabinose induction, suppresses the growth defect of an *E. coli plsC* mutant at non-permissive temperatures in a quantity-dependent manner.

### 3.3. In vivo Substrate Specificity of YihG is Different from that of PlsC

To compare the in vivo substrate specificities of YihG and PlsC, we analyzed the fatty acyl composition of PLs from JC201 cells overexpressing YihG or PlsC grown at 37 °C in the presence of 1% l-arabinose. The *sn*-2 ester bonds of PLs were hydrolyzed by PLA2, and the resulting free fatty acids were analyzed by GC–MS, whereas the resulting lysophosphatidylethanolamines (LPEs) and lysophosphatidylglycerols (LPGs) were analyzed by ESI–MS. As shown in Figure 1C, the palmitoleoyl group (16:1) and the palmitoyl group (16:0) were more abundant in cells overexpressing PlsC, whereas the myristoyl group (14:0) and the *cis*-vaccenoyl group (18:1) were more abundant in cells overexpressing YihG, indicating that YihG preferably introduces 14:0 and 18:1 into the *sn*-2 position of PLs compared with PlsC. As shown in Figure 1D and E, 16:0-LPE and 16:0-LPG were the most abundant LPLs in cells overexpressing PlsC, whereas 18:1-LPE and 18:1-LPG were the most abundant LPLs in cells overexpressing YihG, indicating that YihG preferably introduces fatty acyl groups into the PLs containing 18:1 at the *sn*-1 position compared with PlsC. Thus, we concluded that YihG and PlsC have distinct in vivo substrate specificities.

### 3.4. Deletion of Endogenous YihG Affects Membrane PL Composition

**Figure 2:**
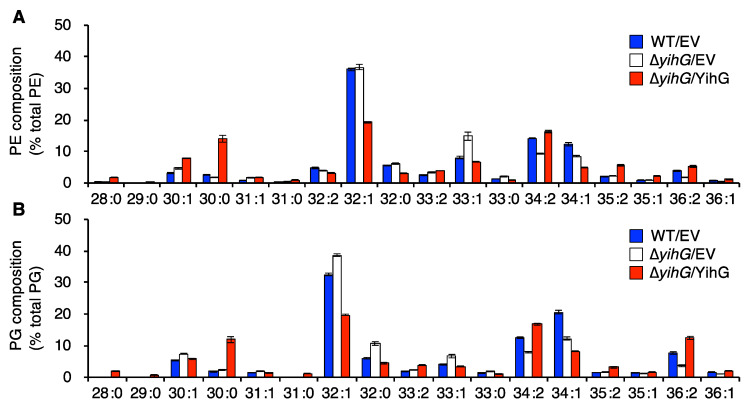
Effects of *yihG* disruption on PL composition. Total PLs extracted from cells grown at 37 °C to an OD_600_ of 0.6–0.8 were analyzed by ESI–MS/MS. The graphs show the composition of PE (**A**) and PG (**B**) derived from wild-type cells harboring the pBAD-Cm^R^ empty vector (WT/EV, blue) and ∆*yihG* cells harboring the pBAD-Cm^R^ empty vector (∆*yihG*/EV, white) or pBAD/*yihG-his_6_* (∆*yihG*/YihG, red). Data are shown as the mean ± SD (*n* = 3).

To investigate the role of endogenous YihG in PL biosynthesis in *E. coli* cells, we analyzed the PL composition of ∆*yihG* cells grown at 37 °C in TB. The PL extracts were subjected to ESI–MS/MS analysis, and the compositions of PE and PG molecular species were determined by their ion peak intensities (Figure 2A,B). The fatty acyl chains in each PL species are summarized in Tables S3 and S4. In ∆*yihG* cells, the levels of 34:2-PE, 34:2-PG, 36:2-PE, and 36:2-PG containing 18:1 decreased compared with those in wild-type cells. The amounts of these PLs were increased when the YihG-expression plasmid was introduced into ∆*yihG* cells. These results suggest that YihG is involved in the biosynthesis of PLs containing 18:1. The amounts of 34:1-PE and 34:1-PG containing 18:1 also decreased in ∆*yihG* cells compared with those in wild-type cells. However, these PLs further decreased when the YihG-expression plasmid was introduced into ∆*yihG* cells. A possible reason for this will be described in the Discussion.

**Figure 3:**
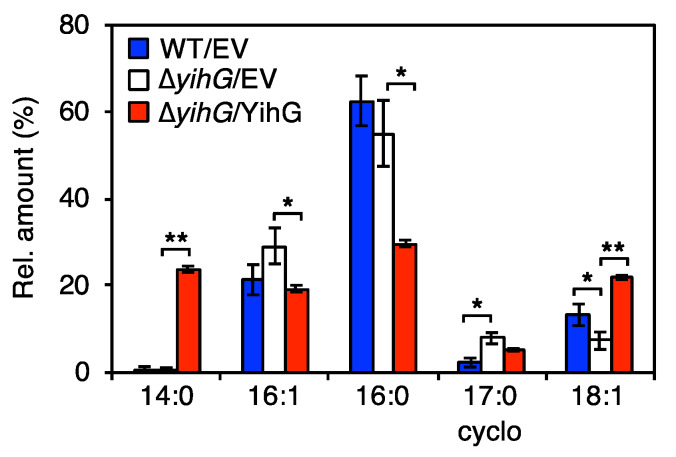
Effects of *yihG* disruption on the fatty acyl group at the *sn*-2 position of PLs. Total PL extracts were hydrolyzed by PLA2, and the resulting fatty acids were analyzed by GC–MS. The graph shows the relative amounts of the fatty acids from wild-type cells harboring the pBAD-Cm^R^ empty vector (WT/EV, blue) and ∆*yihG* cells harboring the pBAD-Cm^R^ empty vector (∆*yihG*/EV, white) or pBAD/*yihG-his_6_* (∆*yihG*/YihG, red). Data are shown as the mean ± SD (*n* = 3). The cyclopropane derivative of 16:1 is indicated as 17:0cyclo. Statistical analysis was performed using Welch’s *t*-test: *, *p* < 0.05; **, *p* < 0.01.

To confirm that endogenous YihG introduces 18:1 into the *sn*-2 position of PLs, we analyzed the *sn*-2 fatty acyl group composition of PLs from ∆*yihG* cells. The *sn*-2 ester bonds of PLs were hydrolyzed by PLA2. The resulting free fatty acids were subjected to GC–MS analysis, and the composition of the fatty acids was determined by their peak intensities (Figure 3). When compared with wild-type cells, the amount of 18:1 linked to the *sn*-2 position significantly decreased in ∆*yihG* cells. This result indicated that YihG introduces 18:1 into the *sn*-2 position of PLs. Consistently, the amount of 18:1 significantly increased when the YihG-expression plasmid was introduced into ∆*yihG* cells. The amount of 14:0 drastically increased when the YihG-expression plasmid was introduced into ∆*yihG* cells. The reason for this could be explained by the substrate preference of YihG for 14:0 (Figure 1C).

### 3.5. The Deletion of Endogenous YihG Causes Enhanced Swimming Motility

To investigate the physiological role of YihG in *E. coli* cells, we characterized the growth phenotype of ∆*yihG* cells. ∆*yihG* cells were cultured in LB and minimal medium at various temperatures. We found that the lack of YihG has no or only marginal effects on the growth of *E. coli* cells under these conditions (Figure S3).

**Figure 4:**
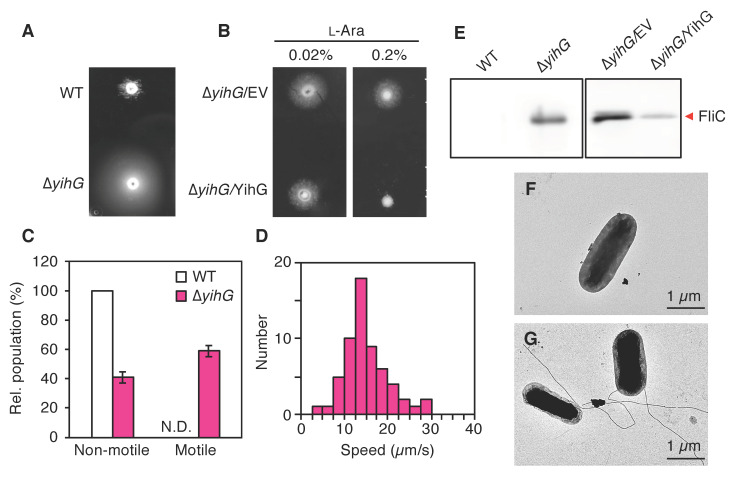
Characterization of the swimming motility of the *E. coli* cells. (**A**) Motilities of wild-type and ∆*yihG* cells on the 0.2% agar plate. The plate was incubated at 37 °C for 12 h. (**B**) Motilities of ∆*yihG* cells harboring the pBAD-Cm^R^ empty vector (∆*yihG*/EV) or pBAD/*yihG-his_6_* (∆*yihG*/YihG) on the 0.2% agar plates containing 0.02% or 0.2% l-arabinose. The plates were incubated at 37 °C for 15 h. (**C**) Motility of the *E. coli* cells in solution. Wild-type (white) and ∆*yihG* (pink) cells were grown at 37 °C to an OD_600_ of 0.6–0.8 and observed under the microscope. Relative populations of motile and non-motile cells were calculated. Data are shown as the average of four biological replicates ± SD. N.D., not detected. (**D**) Histogram of the swimming speed of motile ∆*yihG* cells. (**E**) Effects of *yihG* disruption on the flagellar formation of *E. coli* cells. Flagellin was separated from wild-type and ∆*yihG* cells grown at 37 °C to an OD_600_ of 0.6–0.8 and analyzed by Western blot analysis. Flagellin was also separated from ∆*yihG* cells harboring the pBAD-Cm^R^ empty vector (∆*yihG*/EV) or pBAD/*yihG-his_6_* (∆*yihG*/YihG) grown at 37 °C to an OD_600_ of 0.6-0.8 and analyzed by Western blot analysis. (**F**,**G**) TEM observation of the flagellar structures of the *E. coli* cells. Wild-type (**F**) and ∆*yihG* (**G**) cells were grown at 37 °C on TB agar plates and observed under the TEM.

We subsequently monitored bacterial motility by spotting the cell cultures on TB soft agar plates. *E. coli* BW25113 is known as a motility-impaired strain due to the low transcription level of motility-related genes compared with other motile *E. coli* strains [50]. However, interestingly, ∆*yihG* cells showed a much larger swimming halo compared with wild-type cells after 12 h incubation at 37 °C (Figure 4A), although the growth rate of ∆*yihG* cells was very similar to that of wild-type cells in TB at 37 °C (Figure S4). Motility of ∆*yihG* cells harboring the YihG-expression plasmid was also assayed. These cells were incubated on plates containing 0.02% or 0.2% l-arabinose for 15 h (Figure 4B). The induction of YihG with 0.02% l-arabinose did not affect the motility of ∆*yihG* cells. However, cells overexpressing YihG in the presence of 0.2% l-arabinose did not show the enhanced motility compared with cells harboring the empty vector. These results suggested that YihG is related to the bacterial motility.

We further checked the motility phenotype of ∆*yihG* cells in solution (Figure 4C,D, and Video S1). The cells were grown at 37 °C in TB to an OD_600_ of 0.6 to 0.8 and observed under the microscope at room temperature. Wild-type cells did not show any directional swimming motion but diffused freely in solution. In contrast, more than half of ∆*yihG* cells swam smoothly in solution, and only a small proportion of cells showed a jiggling motion. The fraction of the swimming cells was 59 ± 4% (mean ± SD, four assays), and their speed was 15 ± 5 µm/s (mean ± SD, 59 cells). These results indicated that the motility machinery, the flagellum, functions well in ∆*yihG* cells compared to wild-type cells.

*E. coli* cells swim by rotating flagellar filaments [51,52,53,54], and the speed of swimming cells is dependent on the number of flagellar filaments [55]. Therefore, we analyzed the flagellar expression of wild-type and ∆*yihG* cells. *E. coli* flagellin (FliC) was isolated from the cells and analyzed by Western blot analysis. As shown in Figure 4E, FliC was highly expressed in ∆*yihG* cells compared to wild-type cells. In addition, this phenotype was suppressed by introduction of the YihG-expression plasmid into the mutant and expression of YihG by addition of 0.2% l-arabinose. Finally, the flagellar formation was confirmed by electron microscopy. Wild-type cells showed no evidence of flagellar filaments (Figure 4F). In contrast, approximately half of ∆*yihG* cells had one or two flagellar filaments (Figure 4G). Taking these data together, we concluded that loss of YihG promotes the formation of the functional flagella, leading to the enhanced swimming motility.

## 4. Discussion

To assess the in vivo function of an SlPlsC4 ortholog of *E. coli* named YihG, we conducted a complementation assay using *E. coli* JC201 cells. Overproduction of YihG suppressed the temperature-sensitive phenotype of *E. coli* JC201 carrying a mutated *plsC* (Figure 1A,B and Figure S2), demonstrating that YihG has LPAAT activity. GC–MS analysis of *sn*-2 fatty acyl groups and ESI–MS analysis of 1-acyl LPLs (Figure 1C–E) revealed that YihG facilitates the synthesis of PLs containing 14:0 and 18:1 at the *sn*-2 position and 18:1 at the *sn*-1 position, whereas PlsC facilitates the synthesis of PLs containing 16:1 and 16:0 at the *sn*-2 position and 16:0 at the *sn*-1 position. Thus, the in vivo substrate specificities of YihG and PlsC are clearly different from each other. To our knowledge, this is the first report showing that *E. coli* expresses two functional LPAAT homologs with different substrate specificities. It is notable that deletion of *plsC* is lethal, and that endogenous YihG cannot suppress the growth defect of JC201 cells at non-permissive temperatures. This may be due to low expression of the endogenous YihG. In fact, an increase in the l-arabinose concentration led to a corresponding increase in the growth rate of JC201 cells harboring arabinose-controlled YihG-expression plasmid (Figure 1B).

Next, to unveil the physiological role of endogenous YihG in *E. coli*, we analyzed the effects of *yihG* disruption on PL biosynthesis in *E. coli* BW25113. ESI–MS/MS analysis of PLs (Figure 2) and GC–MS analysis of the *sn*-2 fatty acyl groups (Figure 3) revealed that endogenous YihG plays a major role in the synthesis of PLs containing 18:1 at the *sn*-2 position. However, 18:1 at the *sn*-2 position of PLs still remained in ∆*yihG* cells (levels were approximately a half of that in wild-type cells). Thus, endogenous PlsC also contributes to the synthesis of PLs containing 18:1 at the *sn*-2 position. In fact, PL species containing 18:1 (34:2-PE, 34:2-PG, 34:1-PE, 34:1-PG, 36:2-PE, and 36:2-PG) were still synthesized in ∆*yihG* cells, even though some of these PLs possibly contain 18:1 at their *sn*-1 positions. The amount of some PL species containing 18:1 (34:1-PE and 34:1-PG) further decreased when the YihG-expression plasmid was introduced into ∆*yihG* cells (Figure 2). These results may be due to overexpression of *yihG* under the control of the P_BAD_ promoter [41]. Overproduced YihG is supposed to facilitate incorporation of 14:0 on the acyl carrier protein of fatty acid synthase into 16:0-LPA to produce 30:0-PE and 30:0-PG (Figure 2 and Figure 3) and cause deficiency of 16:0-LPA for the synthesis of other PL species, such as 34:1-PE and 34:1-PG. Consistent with this speculation, the abundance of 32:1-PE and 32:1-PG containing 16:0 also drastically decreased when YihG was overexpressed in ∆*yihG* cells (Figure 2).

Various bacteria, including *N. meningitidis*, *P. fluorescens*, *P. aeruginosa*, *R. capsulatus*, and *S. livingstonensis* Ac10, produce multiple LPAAT homologs [24,25,26,27,32]. However, there is no report describing the occurrence of an LPAAT homolog that preferentially produces PLs containing 18:1 in bacterial cells under physiological conditions. Thus, YihG is a new type of LPAAT homolog that plays a major role in the synthesis of PLs containing 18:1 at the *sn*-2 position under physiological conditions. Although YihG was identified as an ortholog of SlPlsC4 derived from *S. livingstonensis* Ac10, the in vivo substrate specificities of YihG and SlPlsC4 are different from each other. YihG prefers 14:0 and 18:1 as acyl donor substrates (Figure 1C), whereas SlPlsC4 prefers i13:0, 14:0, and i15:0 as acyl donor substrates [34]. This is possibly explained by the differences in the size and shape of their hydrophobic tunnels which accommodate the acyl chains of the acyl-acyl carrier protein or coenzyme A [22]. To understand the molecular basis of substrate specificity of YihG and SlPlsC4, biochemical and structural analyses should be conducted in the future.

In bacteria, PLs that contain low-melting-point fatty acids, such as monounsaturated fatty acids, contribute to the maintenance of membrane fluidity for their optimal growth at low temperatures. In fact, a decrease in monounsaturated fatty acids in the membrane leads to growth defects in some bacteria at low temperatures [7]. However, in the case of *E. coli*, the growth rate of ∆*yihG* cells at 18 °C was barely distinguishable from that of wild-type cells (Figure S3). Thus, PLs containing 18:1 produced by YihG are not essential for the growth of *E. coli* at low temperatures. ∆*yihG* cells still contain a large amount of 16:1 and 18:1, constituting 36.5% of the total fatty acids at the *sn*-2 position of PLs (Figure 3), and this may contribute to the maintenance of the membrane fluidity for the growth of this bacterium.

Interestingly, the deletion of YihG enhanced swimming motility and caused abnormal flagellar production (Figure 4). The expression of motility-related genes is tightly regulated in *E. coli*, and these genes are arranged in hierarchical order into three classes (I, II, and III) [51]. At the top of the hierarchy is the class I operon containing the *flhDC* genes called the master operon. Motile *E. coli* strains like MG1655 and W3110 contain insertion sequence elements IS*1* or IS*5* in the regulatory region of the *flhDC* promoter, and this leads to dramatic activation of the master operon that is otherwise silenced [56,57]. In contrast, poorly motile *E. coli* strains like BW25113 lack such an insertion element in the corresponding region [57,58]. According to this fact, the following reasons may account for the abnormal flagellar production by *E. coli* BW25113 in the absence of *yihG*. First, *flhDC* expression may be derepressed in ∆*yihG* cells due to envelope stress induced by changing membrane PL composition [59], given that a certain bacterial signal transduction system responds to the perturbations in membrane lipid properties [60,61,62,63]. Second, YihG may affect the cellular ratio of DnaA-ATP/DnaA-ADP [40], which not only regulates DNA replication [64,65], but also affects *flhDC* expression [66,67]. An ATP/ADP switch of DnaA defines its DNA-binding activity [68], and this conversion is in part promoted by highly unsaturated membrane PLs [69,70]. Thus, membrane PLs generated by YihG may regulate the cellular DnaA-ATP level, coordinating the DNA replication and the *flhDC* expression. YihG is also conserved in some γ-proteobacteria such as *S. typhimurium* and *V. cholerae*, and flagella play an important role in the adhesion and invasion of these pathogenic cells [71]. We propose that YihG may affect the virulence of these bacteria by regulating flagellar formation according to environmental changes. The details of the mechanism by which YihG affects the flagellar formation should be clarified in the future.

## 5. Conclusions

In this study, we characterized the in vivo function of YihG, a novel *E. coli* LPAAT homolog, and further investigated its physiological roles. Analysis of the fatty acyl composition of PLs from JC201 cells overexpressing YihG and PlsC revealed that YihG facilitates the synthesis of PLs containing 14:0 and 18:1 at the *sn*-2 position and 18:1 at the *sn*-1 position, whereas PlsC facilitates the synthesis of PLs containing 16:1 and 16:0 at the *sn*-2 position and 16:0 at the *sn*-1 position, thus demonstrating that *E. coli* has two functional LPAAT homologs with different substrate specificities. Analysis of the fatty acyl composition of PLs from ∆*yihG* cells revealed that endogenous YihG introduces 18:1 into the *sn*-2 position of PLs. Phenotypic analysis revealed that the lack of YihG causes high expression of FliC and enhances swimming motility but does not affect cell growth or morphology. In addition, the flagellar structure was observed only in ∆*yihG* cells. These results suggested that PlsC is responsible for the synthesis of the majority of membrane PLs, whereas YihG has more specific functions related to flagellar formation via modulation of the fatty acyl composition of membrane PLs.

## Supplementary Materials

The following are available online at https://www.mdpi.com/2218-273X/10/5/745/s1, Figure S1: Multiple sequence alignment of YihG, SlPlsC4, and PlsC; Figure S2: Overexpression of YihG in JC201 cells; Figure S3: Growth characteristics of ∆*yihG* cells in LB and minimal medium; Figure S4: Growth characteristics of ∆*yihG* cells in TB; Table S1: Bacterial strains and plasmids used in this study; Table S2: Sequences of primers used in this study; Table S3: Fatty acyl groups of PEs from wild-type and ∆*yihG* cells harboring the plasmids found in Figure 2A; Table S4: Fatty acyl groups of PGs from wild-type and ∆*yihG* cells harboring the plasmids found in Figure 2B. Video S1: Observation of swimming ∆*yihG* cells in liquid medium.
