# Supplementary material for: A Novel Lysophosphatidic Acid Acyltransferase of Escherichia coli Produces Membrane Phospholipids with a cis-vaccenoyl Group and Is Related to Flagellar Formation
Source: Biomolecules. 2020 May 11;10(5):745. doi: 10.3390/biom10050745 (PMC7277886; doi:10.3390/biom10050745)
Supplement: Supplementary file 1 [file biomolecules-10-00745-s001.zip › Toyotake_Biomolecules_Suppl_rev.pdf]

Supplementary Materials

Table S1. Bacterial strains and plasmids used in this study

Strain or Plasmid	Description	Reference
Strains		
E. coli JC201	*plsC* temperature-sensitive mutant	[35]
E. coli BW25113	*lacI*^{q} *rrnB*$_{T14}$ *ΔlacZ*$_{WJ16}$ *hsdR514* *ΔaraBAD*$_{AH33}$ *ΔrhaBAD*$_{LD78}$	NBRP
E. coli Δ*yihG*	BW25113 Δ*yihG* Ω Km^{R}	NBRP
Plasmids		
pBAD28	Amp^{R}, Cm^{R}; An arabinose-inducible vector	[41]
pBAD-Cm^{R}	Cm^{R}; A ScaI-digested and self-ligated pBAD28	This work
pBAD/*yihG-his*$_{6}$	Cm^{R}; A pBAD-Cm^{R} derivative containing *yihG-his*$_{6}$ at the SalI-HindIII site	This work
pBAD/*plsC-his*$_{6}$	Cm^{R}; A pBAD-Cm^{R} derivative containing *plsC-his*$_{6}$ at the SalI-HindIII site	This work

Table S2. Sequences of primers used in this study

For construction of pBAD/*plsC-his*$_{6}$	
Fw	5′-ATCCTCTAGAGTCGACATGCTATATATCTTTCGTCTTATTATTACCG-3′
Rv	5′-CAAAACAGCCAAGCTTTTAGTGGTGATGGTGATGATGAAGCTTAACTTTTCCGGCGGCTTC-3′
For construction of pBAD/*yihG-his*$_{6}$	
Fw	5′-ATCCTCTAGAGTCGACATGGCTAACCTTTTGAATAAATTC-3′
Rv	5′-CAAAACAGCCAAGCTTTTAGTGGTGATGGTGATGATGAAGCTTCTTATTCTGACGCTGTGATG-3′

Table S3. Fatty acyl groups of PEs from the wild-type and Δ*yihG* cells harboring the plasmids found in Figure 2A

PE	*m/z*	WT/EV and Δ*yihG*/EV	Δ*yihG*/YihG
28:0	634	14:0/14:0, 16:0/12:0	14:0/14:0, 16:0/12:0
29:0	648	N.D.	15:0/14:0, 16:0/13:0
30:1	660	14:0/16:1, 16:0/14:1, 18:1/12:0	16:1/14:0, 16:0/14:1, 18:1/12:0
30:0	662	16:0/14:0	16:0/14:0
31:1	674	15:0/16:1, 14:0/17:0cyclo	15:0/16:1, 17:0cyclo/14:0
31:0	676	16:0/15:0, 14:0/17:0	16:0/15:0, 17:0/14:0
32:2	686	16:1/16:1	16:1/16:1
32:1	688	16:0/16:1	16:0/16:1, 18:1/14:0
32:0	690	16:0/16:0	16:0/16:0
33:2	700	17:0cyclo/16:1	17:0cyclo/16:1
33:1	702	16:0/17:0cyclo	16:0/17:0cyclo
33:0	704	16:0/17:0	16:0/17:0, 19:0/14:0
34:2	714	18:1/16:1	18:1/16:1
34:1	716	16:0/18:1	16:0/18:1
35:2	728	18:1/17:0cyclo, 19:0cyclo/16:1	18:1/17:0cyclo, 19:0cyclo/16:1
35:1	730	18:0/17:0cyclo, 16:0/19:0cyclo, 17:0/18:1, 19:0/16:1	18:0/17:0cyclo, 19:0cyclo/16:0, 18:1/17:0, 19:0/16:1
36:2	742	18:1/18:1	18:1/18:1
36:1	744	18:0/18:1	18:0/18:1

17:0cyclo and 19:0cyclo are the cyclopropane derivatives of 16:1 and 18:1, respectively. The *sn*-1/*sn*-2 fatty acyl groups as estimated from the peak intensities of MS/MS analysis are shown.

Table S4. Fatty acyl groups of PGs from the wild-type and Δ*yihG* cells harboring the plasmids found in Figure 2B

PG	*m*/*z*	WT/EV and Δ*yihG*/EV	Δ*yihG*/YihG
28:0	665	N.D.	14:0/14:0, 16:0/12:0
29:0	679	N.D.	15:0/14:0, 16:0/13:0
30:1	691	14:0/16:1, 14:1/16:0	16:1/14:0, 14:1/16:0, 12:0/18:1
30:0	693	16:0/14:0	16:0/14:0
31:1	705	15:0/16:1	15:0/16:1, 17:0cyclo/14:0
31:0	707	N.D.	16:0/15:0, 17:0/14:0
32:1	719	16:0/16:1	16:0/16:1, 18:1/14:0
32:0	721	16:0/16:0	16:0/16:0, 18:0/14:0
33:2	731	17:0cyclo/16:1, 14:0/19:0cyclo	16:1/17:0cyclo, 19:0cyclo/14:0
33:1	733	16:0/17:0cyclo, 15:0/18:1	16:0/17:0cyclo, 18:1/15:0, 19:0cyclo/14:0
33:0	735	16:0/17:0	17:0/16:0, 19:0/14:0
34:2	745	18:1/16:1	18:1/16:1
34:1	747	16:0/18:1	16:0/18:1
35:2	759	18:1/17:0cyclo, 19:0cyclo/16:1	18:1/17:0cyclo, 19:0cyclo/16:1
35:1	761	18:0/17:0cyclo, 16:0/19:0cyclo, 17:0/18:1, 19:0/16:1	18:0/17:0cyclo, 18:1/17:0, 19:0cyclo/16:0, 19:0/16:1
36:2	773	18:1/18:1	18:1/18:1
36:1	775	18:0/18:1	18:0/18:1

17:0cyclo and 19:0cyclo are the cyclopropane derivatives of 16:1 and 18:1, respectively. The *sn*-1/*sn*-2 fatty acyl groups as estimated from the peak intensities of MS/MS analysis are shown.

```
YihG        MANLLNKFIMTRILAAITLLLSIVLTILVTIFCSVPIIIAGIVKLLLPVPVIWRKVSRFC
SlPlsC4     --------MLAFLPGPILFIISLSLLIINTVVWSTLLTIGGVAKLLLPFLPARQLLTRIM
PlsC        --------------------MLYIFRLIITVIYSILVCVFGSIYCLFSPRNPKHVATFGH

YihG        DFMMYCWCEGLAVLLHLNPHLQWEVHGLEGLSKKNWYLLICNHRSWADIVVLCVLFRKHI
SlPlsC4     NRFMWSWATCNGGILYLISNIEWDVKGLEKLDPNGWYLLISNHVSGFDIAAQTYLLRNHI
PlsC        MFGRLAPLFGLKVECRKPTDAESYGN----------AIYIANHQNNYDMVTASNIVQ---
                                                      Motif I
YihG        PMNKYFLKQQLAWVPFLGLACWSLDMPFMKRYSRAYLLRHPERRGKDVETTRRSCEKFRL
AcPlsC4     PMLKFFLKKELLYVPIMGLGCWALDMPFMDRTSAAKLKKNPKLKGKDLQTTRKACERFKH
PlsC        PPTVTVGKKSLLWIPFFGQLYWLTGNLLIDRNNRTKAHG----------TIAEVVNHFKK
                                      Motif II
YihG        HPTTIVNFVEGSRFTQEKHQQTHSTFQNLLPPKAAGIAMALNVLGKQFDKLLNVTLCYPD
SlPlsC4     MPTSIINYVEGSRFTEEKRLRQNSPYRHLLRPKASGIAFTLSAMGEQFTGLLNITLVYPE
PlsC        RRISIWMFPEGTRSRGRG-----------LLPFKTGAFHAAIAAGVPIIPVCVSTTSN--
                Motif III                                   Motif IV
YihG        NNRQPFFDMLSGKLTRIVVHVDLQPIADELHGDYINDKSFKRHFQQWLNSLWQEKDRLLT
SlPlsC4     TSEKVLTNVMHGRTKKVVVRIETLPVPKVDSEVYFSSPETRVEFQRWLNQLWVEKDQQID
PlsC        -------KINLNRLHNGLVIVEMLPPIDVSQYGKDQVRELAAHCRSIMEQKIAELDKEVA

YihG        SLMSSQRQNK
SlPlsC4     DIIKAYQQQ-
PlsC        EREAAGKV--
```

Figure S1. Multiple sequence alignment of YihG, SlPlsC4, and PlsC. The amino acid sequence of YihG from *E. coli* BW25113 (accession number, AIN34165) was aligned with those of SlPlsC4 from *S. livingstonensis* Ac10 (BBD74888) and PlsC from *E. coli* BW25113 (AIN33369) using the CLUSTALW algorithm on the GenomeNet website (https://www.genome.jp/tools-bin/clustalw). The regions of four conserved acyltransferase motifs described previously [32,39] are underlined, and the key residues in each motif are highlighted in red.

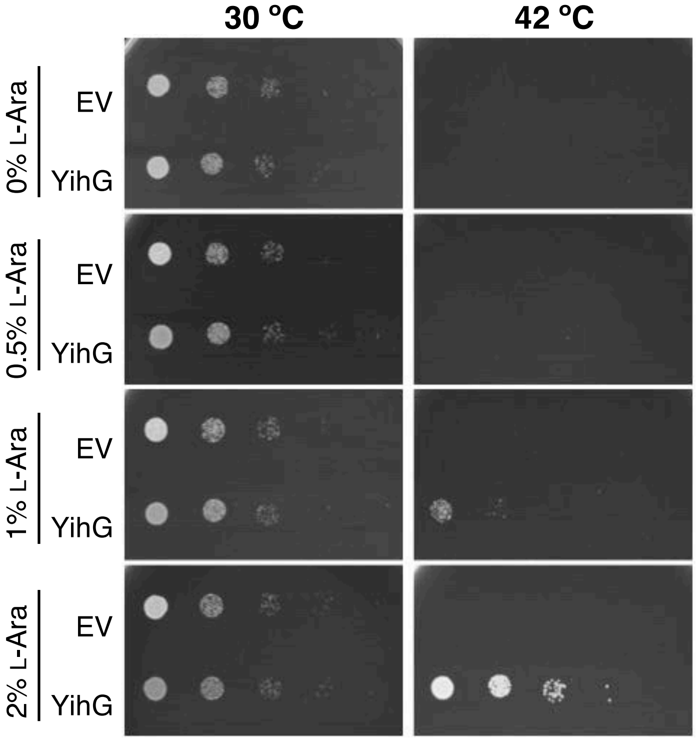

Figure S2. Overexpression of YihG in the JC201 cells. Serially diluted JC201 cells harboring pBAD-Cm^{R} empty vector (EV) or pBAD/*yihG-his*$_{6}$ (YihG) were grown on LB plates containing 0%, 0.5%, 1%, and 2% L-arabinose at 30 °C and 42 °C for 12-14 h.

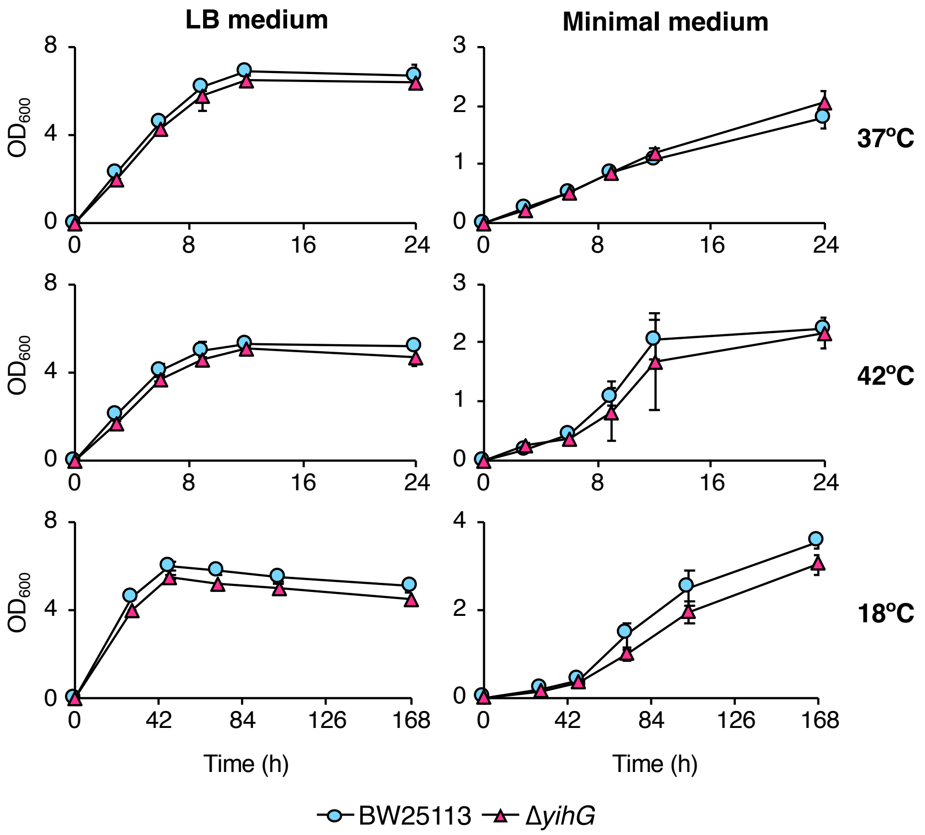

Figure S3. Growth characteristics of the Δ*yihG* cells in LB and minimal medium. The growth of the wild-type (circle, light blue) and Δ*yihG* (triangle, pink) cells at 37 °C, 42 °C, and 18 °C in LB and minimal medium was monitored. Each data point is the average of three biological replicates ± SD.

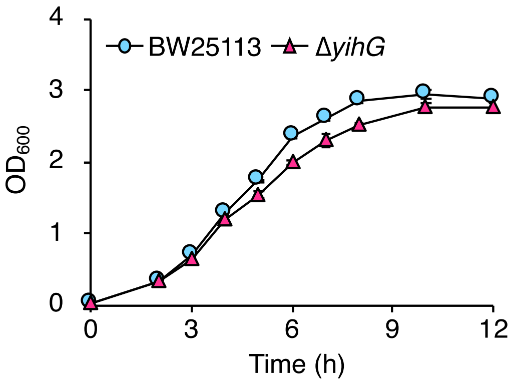

Figure S4. Growth characteristics of the Δ*yihG* cells in TB. The growth of the wild-type (circle, light blue) and Δ*yihG* (triangle, pink) cells at 37 °C in TB was monitored. Each data point is the average of three biological replicates ± SD.

Video S1. Observation of the swimming Δ*yihG* cells in liquid medium. The wild-type (left panel) and Δ*yihG* (right panel) cells were grown at 37 °C in TB. The cultivated cells were diluted with fresh TB medium and observed at room temperature under the microscope.
